# Supplementary material for: Encapsulation of Sorghum Leaf Red Dye: Biological and Physicochemical Properties and Effect on Stability
Source: Foods. 2023 Apr 14;12(8):1646. doi: 10.3390/foods12081646 (PMC10138003; doi:10.3390/foods12081646)
Supplement: Supplementary file 1 [file foods-12-01646-s001.zip › foods-2265112-supplementary.pdf]

Supplementary Materials

Encapsulation of Sorghum Leaf Red Dye: Biological and Physicochemical Properties and Effect on Stability

Carmo Serrano [1,2,*], Margarida Sapata [1], M. Conceição Oliveira [3], Andreia Soares [1], Carla Pereira [4,5], Rui M. V. Abreu [4,5] and Lillian Barros [4,5]

[1] Instituto Nacional de Investigação Agrária e Veterinária (INIAV, I.P.), Av. da República, 2780-157 Oeiras, Portugal
[2] LEAF–Linking Landscape: Environment, Agriculture and Food–Research Center, Instituto Superior de Agronomia, Associated Laboratory TERRA, Universidade de Lisboa, Tapada da Ajuda, 1349-017 Lisboa, Portugal
[3] Centro de Química Estrutural, Institute of Molecular Sciences, Instituto Superior Técnico, Universidade de Lisboa, 1049-001 Lisboa, Portugal
[4] Centro de Investigação de Montanha (CIMO), Instituto Politécnico de Bragança, Campus de Santa Apolónia, 5300-253 Bragança, Portugal
[5] Laboratório Associado para a Sustentabilidade e Tecnologia em Regiões de Montanha (SusTEC), Instituto Politécnico de Bragança, Campus de Santa Apolónia, 5300-253 Bragança, Portugal
* Correspondence: carmo.serrano@iniav.pt

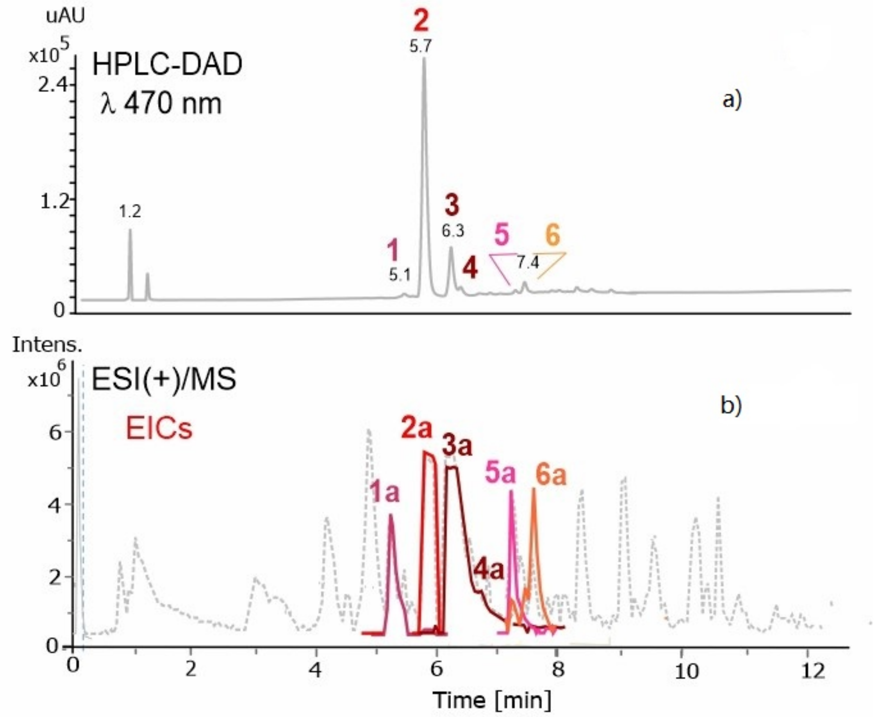

Figure S1. LC-DAD-MS analysis obtained in the ESI negative mode for the *Sorghum bicolor* L. extract: (**a**) DAD chromatogram obtained at 470 nm; (**b**) total ion chromatogram (in gray) and extracted ion chromatograms for the cationic ions of : **1a** *m/z* 271.0601 luteolinidin; **2a** *m/z* 255.0652 apigeninidin; **3a** and **4a** *m/z* 269.0814 methoxyapigeninidin isomers; **5a** *m/z* 509.1238 apigeninidin-flavene dimer, and **6a** *m/z* 523.1392 apigeninidin-7-*O*-methoxyflavene dimer.

The peaks 1-6 observed in Figure S1a have a direct correspondence with peaks 1a-6a observed in Figure S1b being identified trough the EICs values listed in the figure caption.
